# Case report: cystic artery pseudoaneurysm presenting as a massive per rectum bleed treated with percutaneous coil embolization

**DOI:** 10.1186/s42155-019-0090-0

**Published:** 2020-01-15

**Authors:** Frank Carey, Marcus Rault, Michael Crawford, Mark Lewis, Kelvin Tan

**Affiliations:** Interventional Radiology Unit, Norfolk and Norwich University NHS foundation Trust, Norwich, UK

**Keywords:** Cystic artery, Pseudoaneurysm, Embolisation

## Abstract

**Background:**

Cystic artery pseudoaneurysms are rare. It usually occurs as a complication of laparoscopic cholecystectomy, but can arise uncommonly as a complication of acute cholecystitis. Ruptured cystic artery aneurysms present with haemobilia, intraperitoneal or upper gastrointestinal bleeding. We present an unusual case of cystic artery aneurysm presenting as a massive lower gastrointestinal bleed.

**Case presentation:**

A 47-year-old man was admitted with a thoracic abscess and was noted incidentally on CT to have acute cholecystitis. Subsequently the patient then presented with massive fresh PR bleeding. This was found on CT to be the result of a cystic artery pseudoaneurysm with associated gallbladder fistulation to the hepatic flexure, secondary to cholecystitis. The patient was treated with coil embolisation of the cystic artery made a full recovery and was discharged with a view to performing an elective cholecystectomy.

**Conclusion:**

Cystic artery pseudoaneurysm is a rare complication of cholecystitis which can present as massive lower gastrointestinal haemorrhage secondary to cholecystocolic fistula. Percutaneous embolization is a safe and effective treatment in the acute phase.

## Introduction

Cystic artery pseudoaneurysm is a rare occurrence, most often arising as a complication of biliary procedures. This is most commonly laparoscopic cholecystectomy (Kaman et al. 1998), however cases of ruptured cystic artery pseudoaneurysms have been described following endoscopic retrograde cholangiopancreatography (ERCP) (Proença et al. 2019). Less commonly they can arise as a consequence of acute cholecystitis (Nkwam and Heppenstall 2010), in patients with polyarteritis nodosa (Saluja et al. 2007), and abdominal trauma (Anand et al. 2011). Clinical presentation is usually associated with rupture, which occurs in 45% of cases, leading to haemorrhagic cholecystitis, intraperitoneal bleeding and upper GI bleeding (Anand et al. 2011). Patients may present with Quincke’s triad of right upper quadrant pain, upper gastrointestinal bleeding and jaundice (Alis et al. 2016). Unruptured pseudoaneurysms are occasionally detected incidentally during CT investigation. To our knowledge there are no previous cases in literature with fresh PR bleeding occurring due to a cholecystocolic fistula arising secondary to gallbladder inflammation.

## Case report

**Fig. 1:**
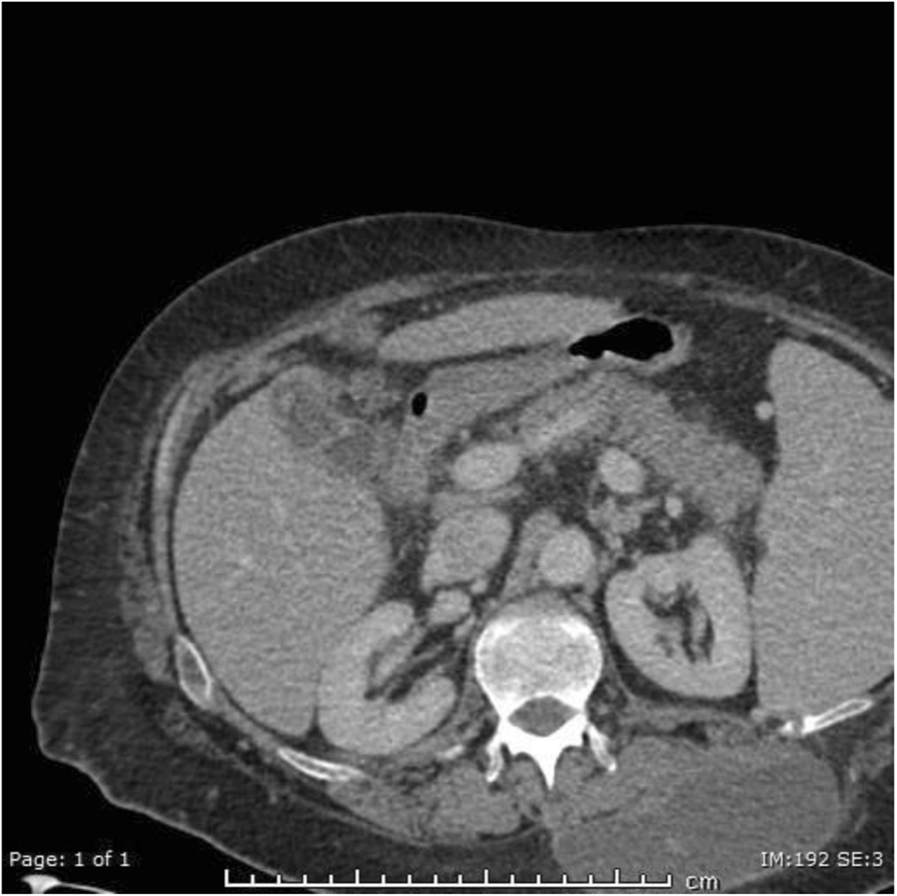
Pre-contrast CT. Gallbladder wall thickening

A 47-year-old man was admitted to hospital with a 9 day history of abdominal pain and diarrhoea and an incidental fluctuant swelling on his back. He had a previous medical history of alcoholic liver cirrhosis, anaemia and hypothyroidism. Examination revealed a fluctuant tender swelling on the left side of his back extending to the inferior angle of scapula. His blood tests demonstrated raised inflammatory markers (WCC 12.3, CRP 169, normal LFTs). On CT he was found to have a large left pleural enhancing collection with adjacent rib destruction corresponding to the clinically palpable mass. At that time note was made of incidental mild acute cholecystitis (Fig. 1). The patient required rib resection, drainage of his pleural empyema and antibiotics.

**Fig. 2:**
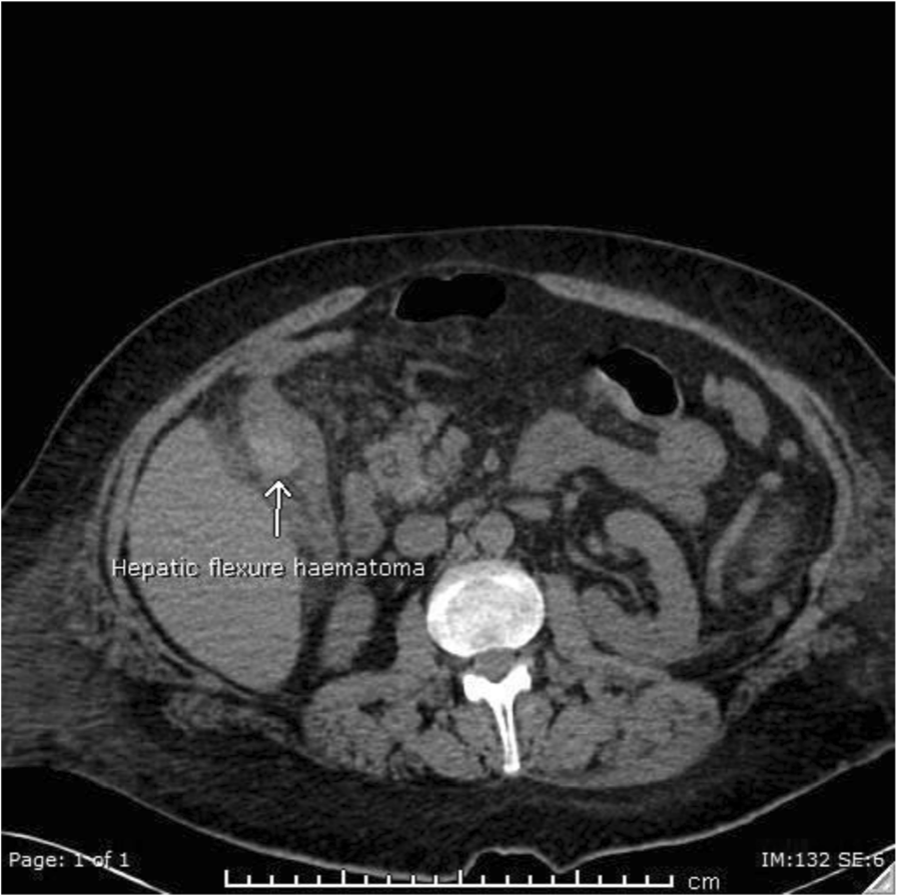
Pre-contrast CT. Dense fluid within the hepatic flexure

**Fig. 3:**
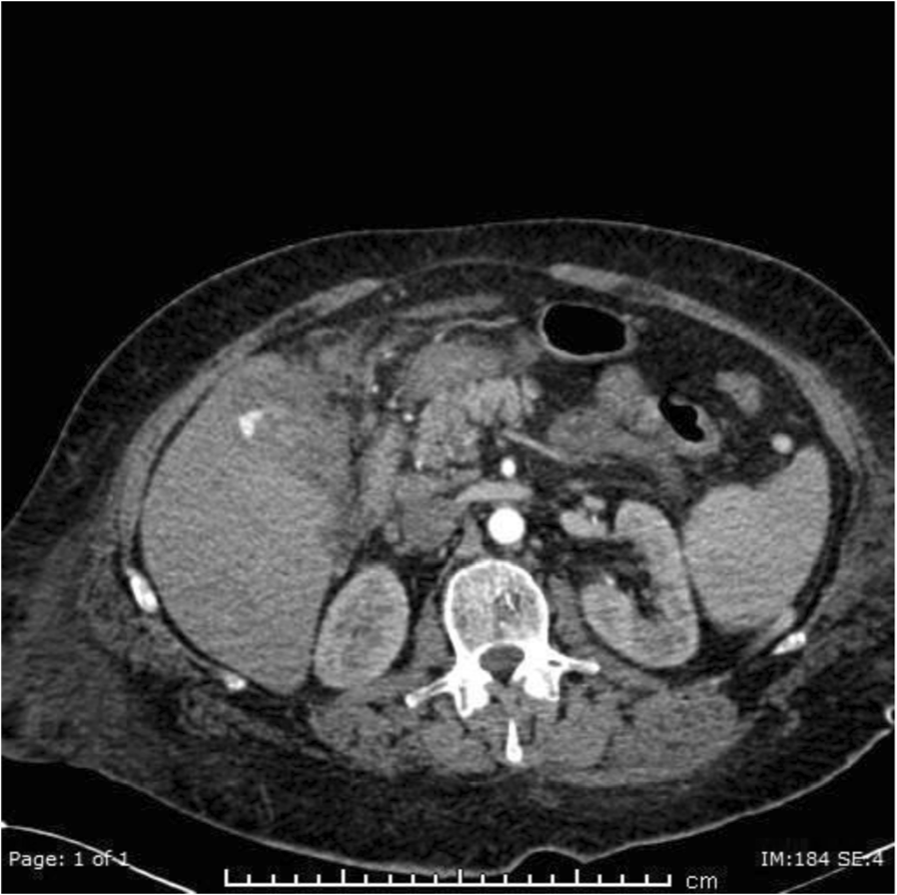
Arterial phase CT. Contrast blush within the gallbladder

**Fig. 4:**
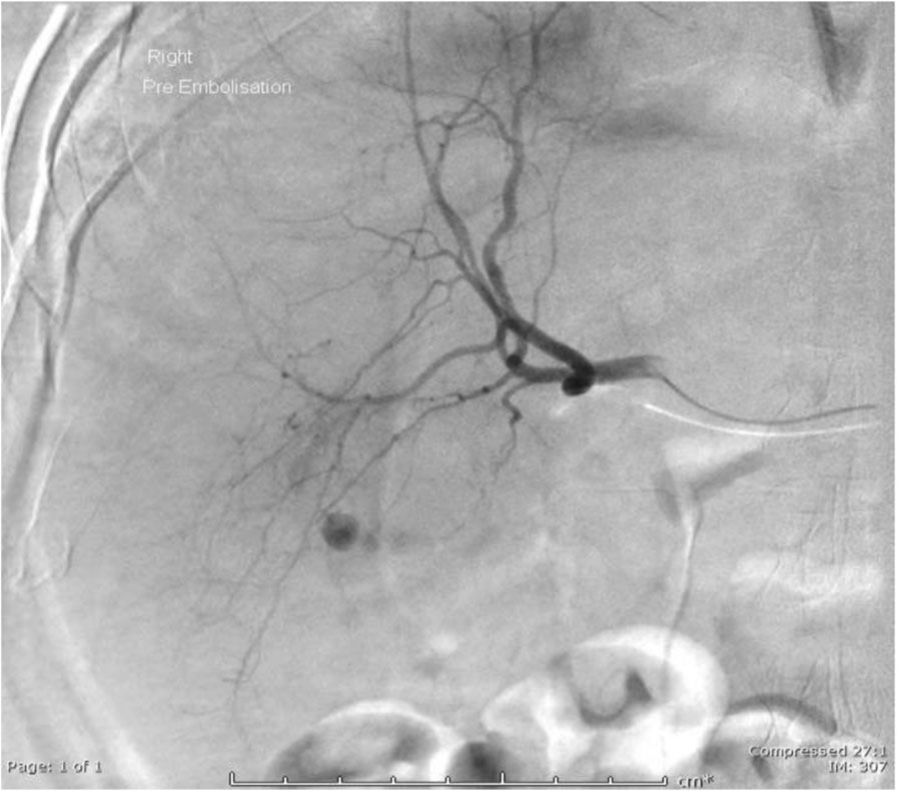
Hepatic artery angiogram demonstrating cystic artery pesudoaneurysm

**Fig. 5:**
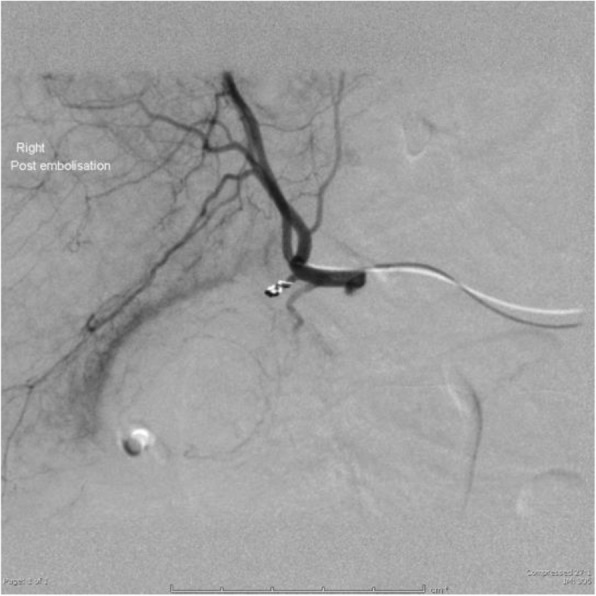
Post successful cystic artery embolisation

On day 7 post op he suffered a massive fresh PR bleed. Hb measured 61 g/L having been 103 g/L on admission. There was no evidence of coagulopathy and liver function tests were unremarkable. The patient was treated with blood transfusion, FFP and tranexamic acid. Flexible sigmoidoscopy revealed no cause for fresh PR bleeding. Triple phase CT was performed. The pre-contrast images demonstrated layering of variable density fluid in the gallbladder with the hyperdense fluid also within the colon at the hepatic flexure, which was located adjacent to and inseparable from the gallbladder (Fig. 2). On the post-contrast images there was a blush within the gallbladder in keeping with a cystic artery pseudoaneurysm (Fig. 3). The patient was transferred to IRU where DSA confirmed the cystic artery pseudoaneurysm (Fig. 4). This was embolised with a single 2 × 3 mm Tornado coil (Cook Medical) (Fig. 5). Ideally the coil would have been deployed more distally relative to the origin of the cystic artery, however despite prolonged attempts, the microcatheter would not advance distally and so the coil was deployed as demonstrated.

Following embolization, the patient was transferred to ITU but suffered no further episodes of bleeding. Repeat CT scan demonstrated successful embolization of the cystic artery pseudoaneurysm. The patient was stepped down to the ward 2 days later and discharged 1 week after embolization on oral antibiotics for his pleural empyema with a surgical outpatient appointment regarding future cholecystectomy.

## Discussion

Cystic artery pseudoaneurysms are rare. Most commonly they arise as a complication of cholecystectomy. They can also occur secondary to acute cholecystitis. The pathophysiology of cholecystitis related pseudoaneurysm formation is thought to be a consequence of inflammation and fibrosis causing weakness in the artery wall and resultant pseudoaneurysm formation (Alis et al. 2016). Patients usually present with haemobilia, haemoperitoneum or rarely upper gastrointestinal bleeding. Cystic artery pseudoaneurysms can be detected on US where there may be hyperechoic fluid in the gallbladder if the pseudoaneurysm has ruptured, and colour Doppler may show the “Yin-Yang” flow within the pseudoaneurysm itself. Triple phase CT angiography is sensitive for detecting pseudoaneurysms, often also identifying the underlying cause (for example acute cholecystitis), and is helpful in planning percutaneous treatment especially identifying abnormal vessels and anatomic variations. Previously patients with cystic artery pseudoaneurysms would undergo open surgical repair rather than laparoscopic repair due to the risk of rupture (Loizides et al. 2015). Percutaneous selective cystic artery embolization is shown to be an effective treatment strategy in these patients in the acute setting with less mortality and morbidity than surgery with better identification of the bleeding vessel and higher rates of achieving haemorrhagic control (Lygidakis et al. 1991) Options for embolization include coils, glue injection or Gelfoam (Pfizer) injection. Coil embolization is the favoured technique as coils can be used to treat a variety of vessel sizes and can be introduced without the risk of increasing the pressure in this vascular lesion, which may be a risk with the injected embolic agents such as glue or Gelfoam (Desai and et al. 2010). Cystic artery embolisation has been shown to be a safe procedure. Non-target embolisation of hepatic parenchymal tissue can expose the patients to the rare risks associated with any hepatic artery embolization such as ischaemic hepatitis and abscess formation, and patients should be monitored for this post-procedure (Clark et al. 2006). However, the superselective nature of cystic artery embolisation should reduce the risk of these side effects and none were reported in the literature. On searching the literature, we were unable to find a similar case where the patient presented with a fresh PR bleed secondary to a cholecystocolic fistula which underwent successful endovascular embolization treatment.

## Conclusion

Cystic artery pseudoaneurysms are rare complications of acute cholecystitis which usually presents with pain, haemobilia and or upper gastrointestinal tract bleeding. We present a rare case of fresh PR bleeding secondary to a cholecystocolic fistula which was successfully treated with coil embolisation.

## Data Availability

The data used during the study are available from the corresponding author on request.

## Abbreviations

CAP: Cystic artery pseudoaneurysm
CRP: C reactive protein
DSA: Digital subtraction angiography
FFP: Fresh frozen plasma
WCC: White cell count
